# HTLV-I Basic Leucine Zipper Factor (sHBZ) Actively Associates with Nucleophosmin (B23) in the Nucleolus

**DOI:** 10.3390/v17050727

**Published:** 2025-05-19

**Authors:** Nahid Moghadam, Yong Xiao, Francois Dragon, Benoit Barbeau

**Affiliations:** 1 Department of Biological Sciences, University of Quebec at Montreal (UQAM), 141 President-Kennedy Avenue, Montreal, QC H2X 3X8, Canada; moghadam.nahid@courrier.uqam.ca (N.M.); xiao.yong@uqam.ca (Y.X.); dragon.francois@uqam.ca (F.D.); 2 Centre d’Excellence en Recherche sur les Maladies Orphelines-Fondation Courtois, 141 President-Kennedy Avenue, Montréal, QC H2X 3X8, Canada; 3 Réseau Communautés Rurales et Éloignées en Santé, 385 Sherbrooke East, Montreal, QC H2X 1E3, Canada

**Keywords:** HTLV-1, ATL, sHBZ, nucleolus, T cell proliferation, nucleophosmin/B23

## Abstract

Human T cell leukemia virus type 1 (HTLV 1) is an oncogenic retrovirus responsible for the development of adult T cell leukemia (ATL). The minus strand of HTLV-1 provirus encodes an oncoprotein named HTLV-1 bZIP factor (HBZ), which plays a pivotal role in viral replication and T cell proliferation. Of particular interest is the spliced HBZ isoform (sHBZ), which is predominantly expressed in ATL cells and localizes within the nucleolus, conferring immortalizing properties to T cells. Our previous study has shown that sHBZ colocalizes and associates with Nucleophosmin/B23, a nucleolar phosphoprotein with multiple functions. In this study, through an optimized nucleolar isolation method, we first confirmed sHBZ’s nucleolar localization via Western blotting in transfected HEK293T cells, chronically HTLV-1-infected T cell lines, and freshly infected HeLa cells. We further demonstrated that the sHBZ/B23 association predominantly occurs in the nucleolus by co-immunoprecipitation of cell fractions. Our study highlights the nucleolar localization of sHBZ and its possibly essential interaction with this nucleolar-residing protein, leading to cell immortalization.

## 1. Introduction

Human T cell leukemia virus type 1 (HTLV-1) was the first discovered human retrovirus which remains a significant global health concern due to its association with severe diseases and its persistent nature [1]. HTLV-1 infection is estimated to affect 10–20 million people worldwide, with a broad geographic distribution including endemic regions in Japan, the Caribbean, South America, Sub-Saharan Africa, and parts of the Middle East and Central Australia [2]. Despite its widespread prevalence, the majority of infected individuals remain asymptomatic throughout their lives. However, approximately 5–10% of HTLV-1 carriers develop serious diseases, such as adult T cell leukemia/lymphoma (ATL), an aggressive malignancy of CD4^+^ T cells, and HTLV-1-associated myelopathy/tropical spastic paraparesis (HAM/TSP), a debilitating neuroinflammatory condition [3,4]. HTLV-1 has a remarkable ability to establish persistent infections in the host after the integration of its genome into the cellular DNA of infected cells, which plays a critical role in HTLV-1 pathogenesis [5]. The progression from an asymptomatic carrier status to a disease status is believed to be driven by a complex interplay of host immune responses, genetic factors, and the activity of viral proteins [6].

Two viral proteins, Tax and HBZ (HTLV-1 bZIP factor), are central to HTLV-1-mediated pathogenesis [7]. Tax plays a critical role in the early stages of infection by activating viral replication and altering host immune responses. However, its immunogenic nature leads to its suppression in later stages of infection, leaving HBZ as the main driver of disease progression and cellular transformation [8]. Unlike Tax, HBZ is transcribed from the antisense strand of the HTLV-1 genome [9,10] and is unique among viral proteins in being expressed constitutively, even during latency [7,11]. This persistent expression highlights its critical role in viral persistence and disease progression [12].

HBZ is a nuclear protein composed of the N-terminal activation domain, two basic regions (BR1 and BR2), and a DNA-binding domain (DBD) preceding its leucine zipper [10]. Through its basic Zip (bZIP) domain, it interacts with cellular bZIP proteins, such as cAMP-response element binding proteins (CREB, CREB-2), the cAMP-responsive element modulator (CREM-Ia) [10], the activating transcription factor (ATF-3) [13], c-Jun, JunB [14], and JunD [15]. In addition, HBZ suppresses Tax-mediated viral transcription [16] and inhibits the classical nuclear factor-kappa B (NF-κB) pathway by preventing p65 DNA binding and promoting its degradation. This inhibition weakens the host’s antiviral response, facilitating immune evasion and enhancing viral persistence [17]. Furthermore, HBZ regulates key tumor suppressor proteins, including p53 [18] and pRb [19]. By interacting with p53, HBZ suppresses its transcriptional activity, thereby inhibiting apoptosis and promoting cell survival [18]. HBZ affects pRb function by modulating the activity of the E2F transcription factor, a crucial regulator of cell cycle progression. These interactions contribute to the immortalization and transformation of infected T cells [19].

HBZ exists in two isoforms, the spliced (sHBZ) form containing 206 amino acids and the unspliced (usHBZ) form with 209 amino acids, which share 95% amino acid sequence identity and differ in their N terminus [20,21]. sHBZ is the predominant isoform and has been uniquely linked to the induction of IL-2-independent T cell proliferation [22,23]. Interestingly, the subnuclear distribution of these isoforms also differs significantly, with sHBZ primarily localized in the nucleolus [20], the sub-nuclear compartment associated with ribosome biogenesis, as well as roles in cell cycle regulation, stress response, and apoptosis [24,25].

Our research has focused on elucidating the relationship between the nucleolar localization of sHBZ and its role in promoting T cell proliferation and transformation. As proteins residing within the nucleolus have been found to actively participate in cellular transformation processes [26], we hypothesized that its interaction with nucleolar proteins might be central to its potential for immortalization and transformation. In our previous study, we showed that HBZ associates and co-localizes with nucleophosmin (B23) [27], a nucleolar factor which is a regulator of cell proliferation. B23 is a multifunctional phosphoprotein that plays crucial roles in ribosome biogenesis, genome stability, and the regulation of tumor suppressor proteins such as p53 and pRb [28]. The association of sHBZ with B23 suggests a potential mechanism through which HTLV-1 could manipulate host cellular functions to promote viral persistence, evade immune responses, and drive oncogenesis.

In this study, we aimed to determine the cellular compartment where the sHBZ-B23 association occurs. Using a nucleolar isolation method, we analyzed sHBZ’s localization in different cellular models and demonstrated that sHBZ predominantly associates with B23 in the nucleolus. These findings provide critical insights into the role of nucleolar localization in HTLV-1-associated pathogenesis, offering a foundation for future exploration of targeted therapeutic strategies.

## 2. Materials and Methods

### 2.1. Culture of Cell Lines

Human Jurkat E6.1, chronically HTLV-1-infected MT2, and C8166-45 T cell lines were provided by the Biological and Emerging Infections (BEI) Research Resource Program and cultured in RPMI 1640 medium supplemented with 10% fetal bovine serum (FBS) and 1% *v*/*v* penicillin/streptomycin antibiotic solution at 37 °C under 5% CO_2_ atmosphere. HEK293T and HeLa cell lines were obtained from the American Type Culture Collection and cultured in Dulbecco’s Modified Eagle Medium supplemented with 10% FBS and antibiotics at 37 °C under 5% CO_2_ atmosphere.

### 2.2. Plasmids

Expression vectors pcDNA3.1 myc His-sHBZ, GFP-sHBZ, and GFP-usHBZ were kindly provided by J.M. Mesnard (Université de Montpellier 1, Montpellier, France) [21,29]. Additionally, the pGFP-APH-2 vector was generously given by R. Mahieux (ENS Lyon, France).

### 2.3. HTLV-1 Infection of HeLa Cells by Coculture with MT2 Cells

Following a previously established protocol [30], HeLa cells (1 × 10^6^ cells) were co-cultured with 1 mL of MT2 cell suspension (3 × 10^6^ cells/mL) in the presence of 8 µg/mL of polybrene. After a 72 h incubation at 37 °C, MT2 cells were removed by extensive shaking and subsequent washing with PBS. Fresh complete DMEM medium was added to HeLa cells and incubated for an additional 24 h.

### 2.4. Cell Transfection

HEK293T (3 × 10^6^) and HeLa (2 × 10^4^) cells were seeded 24 h prior to transfection using the polyethyleneimine (PEI) reagent (5 µg) and 0.5 µg of DNA. After 12–16 h, cells were washed and supplemented with fresh DMEM. Cells were analyzed 48 h after transfection.

### 2.5. Nucleolus Isolation and Cell Fractionation

Cell fractions were prepared from HeLa, HEK293T, and C8166-45 T cells using a protocol previously described [31]. HeLa and HEK293T cells were grown in 10 × 14 cm Petri dishes until they reached over 90% confluence (approx. 10^7^ cells per dish). C8166-45 T cells were expanded to a density of approximately 1 × 10^8^ cells in total. All solutions described below contained a protease inhibitor cocktail. Cells were first harvested and washed three times with PBS by centrifugation at 228× *g* for 5 min at 4 °C. Cell pellets were then resuspended in 5 mL Buffer A (10 mM Hepes, pH 7.9, 10 mM MgCl_2_, 0.5 mM DTT), and the suspension was incubated on ice for 15 min. Cell suspensions were subjected to Dounce homogenization, with a total of 10 × 10 strokes using a tight pestle. After every 10 strokes, the homogenate was examined under a phase contrast microscope until cell lysis was over 90%. A small aliquot of the homogenate was collected for whole lysate analysis, while the remainder was centrifuged at 218× *g* for 5 min at 4 °C to obtain a nucleus-enriched pellet. The pellet was resuspended in 3 mL S1 solution (0.25 M sucrose, 10 mM MgCl_2_) and layered over 3 mL S2 solution (0.35 M sucrose, 0.5 mM MgCl_2_), followed by centrifugation at 1430× *g* for 5 min at 4 °C. The resulting nuclear pellet was resuspended in 3 mL S2 solution and subjected to sonication using a XL 2020 sonicator (Misonix Inc., Farmingdale NY, USA) equipped with a microtip probe. Sonication was performed in six 10 s bursts on ice, with 10 s intervals between bursts, set at a power level of 5. The sonicated mixture was visually inspected under a phase-contrast microscope to confirm the absence of intact cells while ensuring that nucleoli appeared as dense, refractile bodies. A small portion of the sonicated preparation was collected as the nuclear fraction. The remainder was layered over 3 mL S3 solution (0.88 M sucrose, 0.5 mM MgCl_2_) and centrifuged at 3000× *g* for 10 min at 4 °C. The supernatant was identified as the nucleoplasmic fraction, while the pellet contained nucleoli. The pellet was resuspended in 0.5 mL S2 solution and subjected to a second centrifugation at 1430× *g* for 5 min at 4 °C to obtain purified nucleoli. To extract nucleolar proteins, nucleoli were resuspended in 0.2 mL high-salt RIPA buffer containing 8 mg/mL DNase I and incubated on ice for 30 min. Subsequently, the mixture was sonicated using the same conditions described above (power setting of 3). The homogenate was then centrifuged at high speed (13,000× *g*) for 10 min at 4 °C to separate soluble proteins from insoluble debris. The supernatant was collected, and 0.467 mL low-salt RIPA buffer was added. Protein concentration was determined using the Pierce ^TM^ BCA Protein Assay Kit (Thermo Fisher Scientific IncInc, Montreal, QC, Canada), following the manufacturer’s instructions.

### 2.6. Western Blot Analysis

Proteins from each cell fraction were separated by 12% SDS-PAGE and transferred to a Amersham PVDF-blotting membrane (Amersham, Cytiva Life Sciences, Malborough, UK). Membranes were blocked in PBS/5% milk or PBS/0.3% BSA and incubated with one of the following antibodies in PBS containing 5% skim milk powder and 0.1% Tween-20: mouse monoclonal anti-fibrillarin (1:1000, #sc-166001), mouse monoclonal anti-nucleoporin NUP62 (1:500, #sc-48389) (Santa Cruz Biotechnology), mouse monoclonal anti-α-Tubulin (1:5000, #T5168, Sigma-Aldrich, Saint Louis, MO, USA), mouse monoclonal anti-HBZ (1:800, a kind gift of J.M. Mesnard [10]), mouse monoclonal anti-GFP (1:1000, #sc-9996 HRP), mouse monoclonal anti-myc (1:1000, #sc-40) (Santa Cruz Biotechnology), or rabbit polyclonal anti-B23 (1:2000, #10306-1-AP, Proteintech, Rosemont, IL, USA). After several washes with PBST, membranes were incubated with HRP-conjugated sheep anti rabbit IgG (1:5000, #5220–0336) or anti-mouse IgG (1:5000, #5220–0338) (Seracare, Milford, MA, USA) antibodies in PBS containing 5% skim milk powder and 0.1% Tween-20 for 2 h, washed several times, and incubated with BM Chemiluminescence Blotting Substrate. Membranes were analyzed using the Fusion FX7 device (MBI, Montreal, QC, Canada).

### 2.7. Co-Immunoprecipitation Experiments

Immunoprecipitation was conducted using Dynabeads Protein G (Invitrogen, Ottawa, ON, Canada) following manufacturer’s instructions. Briefly, 25 μL of Dynabeads Protein G was incubated 2 h at room temperature on an orbital rotator with 5 μg anti-myc or 4 μg anti-HBZ antibodies in 300 μL PBS. Equal portions of each fraction, which reflected the same initial number of cells, were added to the antibody-bound beads and incubated overnight at 4 °C with gentle rotation. Bound proteins were eluted following addition of 50 μL of 4X-SDS protein sample buffer containing 50 mM 1,4-dithiothreitol (DTT), followed by heating at 95 °C for 5 min. The eluted samples were resolved by 12% SDS-PAGE and analyzed by Western blot using anti-B23, anti-myc, and anti-HBZ antibodies.

### 2.8. Immunofluorescence

HeLa cells were grown on chamber glass slides (2 × 10^4^ cells/well) and transfected with expression vectors for myc-tagged or GFP-fused sHBZ, as described above. Forty-eight hours after transfection, cells were fixed with 4% paraformaldehyde (PFA), permeabilized with PFA 4% containing 0.1% Triton X-100, and incubated overnight with anti-B23, anti-C23 (1:500; #sc-13057, Santa Cruz Biotechnology, Dallas, TX, USA) and anti-myc antibodies. After three washes in cold PBS, cells were subsequently incubated with goat anti-mouse IgG coupled to Alexa Fluor 568 (#A11004), goat anti-rabbit IgG coupled to Alexa Fluor 488 (#A11008), and goat anti-rabbit IgG coupled to Alexa Fluor 594 (#A11012) (Invitrogen) (1:1000 dilution) for 1 h at room temperature. After three washes in cold PBS, nuclei were counterstained using 4′,6′-diamidino-2-phenylindole (DAPI) for five min. Slides were then mounted in ProLong Antifade reagent. Samples were observed at room temperature with a 60× objective under oil immersion and with a numerical aperture (NA) of 1.4 using a Nikon A1 laser scanning confocal microscope (Nikon Canada, Mississauga, ON, Canada).

## 3. Results

### 3.1. Isolation of Nucleolar Fraction

**Figure 1:**
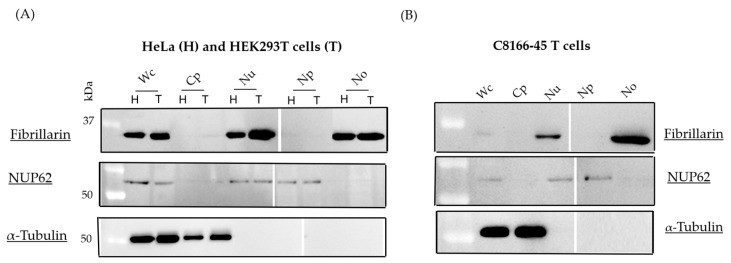
Efficiency of the nucleolar extraction method. Nucleoli were isolated from HeLa (H) and HEK293T (T) cells (**A**) and C8166-45 T cells (**B**). Proteins from subcellular fractions were separated by 12% SDS-PAGE and probed with anti-α-tubulin (cytoplasmic), anti-NUP62 (nucleoplasmic), and anti-fibrillarin (nucleolar) antibodies. Each lane was loaded with a fraction sample derived from the same number of cells. These results are representative of three independent experiments. Wc, whole cell; Cp, cytoplasm; Nu, nuclei; Np, nucleoplasm; No, nucleoli.

In order to clearly demonstrate the sHBZ-B23 association in the nucleoli, highly purified preparations were needed as a source material for our analyses. Nucleoli were first isolated from HeLa and HEK293T cells, and their purity was confirmed using light microscopy.To convincingly address the purity of the different fractions (including the nucleoli), protein samples from whole lysate, cytoplasm, unfractionated nuclear extract, nucleoplasm, and nucleoli were analyzed by Western blotting with antibodies specific for either tubulin, NUP62, and fibrillarin (Figure 1A). As shown in Figure 1A, fibrillarin was highly enriched in the nucleolar fraction. In contrast, NUP62 was more abundant in the nucleoplasm and total nuclear extract. As expected, tubulin exclusively localized to the cytoplasm. Moreover, when the nucleolar isolation protocol was applied to C8166-45 T cells, these fraction markers again demonstrated that cellular fractions were adequately isolated (Figure 1B).

### 3.2. Subcellular Localization of sHBZ Was Maintained During Nucleolus Isolation

**Figure 2:**
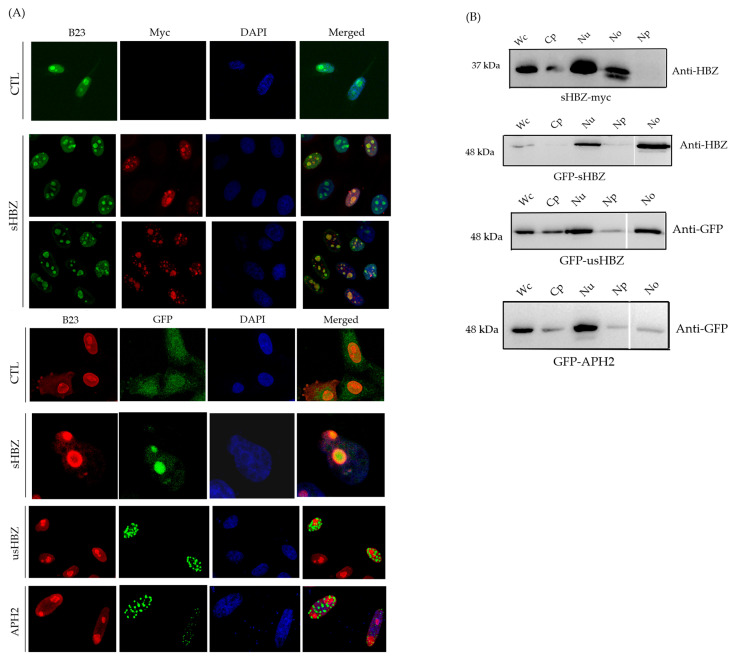
Subcellular distribution of sHBZ. (**A**) HeLa cells were transfected with expression vectors for myc-tagged sHBZ (vs. pcDNA3.1) and GFP-fused sHBZ (vs. GFP), usHBZ, and APH2. Fixed cells were analyzed by confocal microscopy using anti-B23 and/or anti-myc antibodies following staining with DAPI. (**B**) HEK293T cells were transfected with expression vectors for myc-tagged sHBZ and GFP-fused sHBZ, usHBZ, and APH2. Proteins from the different subcellular fractions were analyzed by Western blot with anti-HBZ or anti-GFP, antibodies. Each lane was loaded with a fraction sample derived from the same number of cells. These results are representative of three independent experiments. Wc, whole cell; Cp, cytoplasm; Nu, nuclei; Np, nucleoplasm; No, nucleoli.

We next wanted to evaluate which cell fractions were indicative of sHBZ abundance. Consistent with previous studies [20], our confocal microscopy analyses conducted on transfected HeLa cells expressing myc-tagged or GFP-fused sHBZ (Figure 2A; Supplementary Figure S1) confirmed the colocalization of B23 and sHBZ, as well as of C23 and sHBZ, further indicative of sHBZ’s nucleolar localization. Importantly, Western blot analysis of cell fractions revealed a prominent sHBZ signal in the nucleolar fraction (Figure 2B). In contrast, the unspliced isoform of HBZ (usHBZ) and the Antisense Protein of HTLV-2 (APH-2), the HBZ counterpart in the non-leukemogenic HTLV-2 virus, were more importantly detected in the cytoplasm with a lesser abundance in the nucleolar fraction (Figure 2A,B), as demonstrated by both Western blot analyses of cell fractionation and confocal microscopy, consistent with prior observations [20,32].

### 3.3. The sHBZ-B23 Association Occurs Predominantly in the Nucleolus

**Figure 3:**
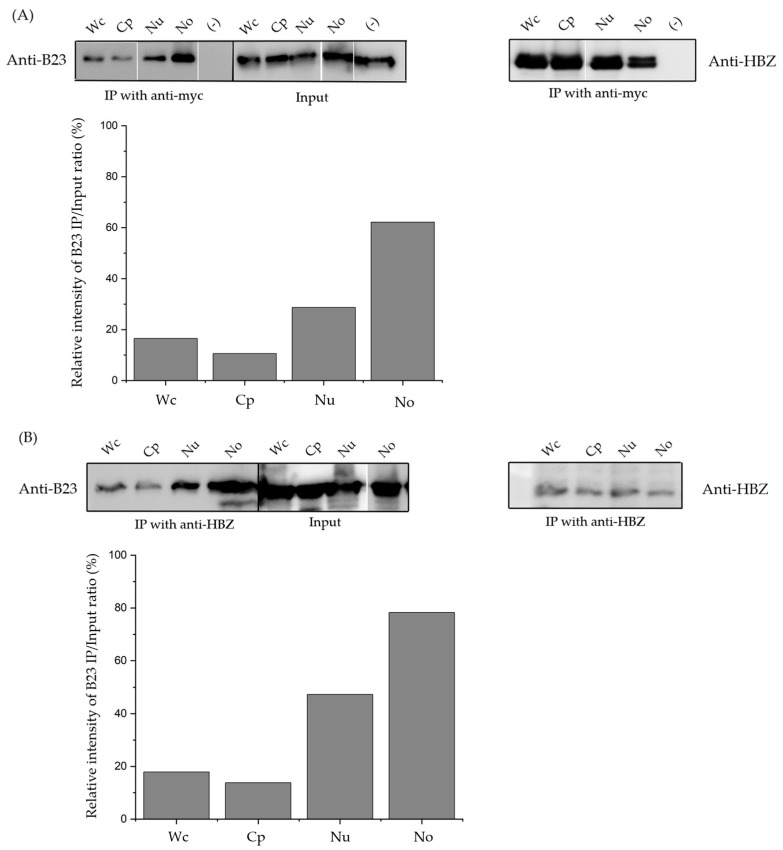

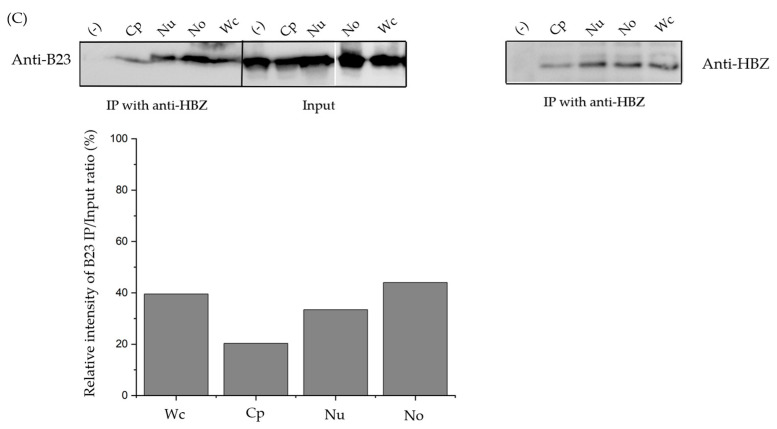
sHBZ-B23 association occurs predominantly in the nucleoli. HEK293T cells were transfected with expression vectors for myc-tagged sHBZ and/or empty vectors as a negative control (-) (**A**). Transfected HEK293T (**A**), C8166-45 (**B**), and non-infected HeLa (-) and infected HeLa cells (**C**) were subjected to nucleolar isolation. Cellular fractions were co-immunoprecipitated with anti-myc or anti-HBZ antibodies. Immunoprecipitates or cellular fractions were analyzed by Western blotting using anti-B23 or anti-HBZ antibodies. The intensity of B23 signals was quantified by densitometric analyses using ImageJ 1.54f. The negative control (-) in C represents HeLa cell cultures in the absence of MT2 cells. Wc, whole cell; Cp, cytoplasm; Nu, nuclei; Np, nucleoplasm; No, nucleoli.

In our previous study, we demonstrated that sHBZ is associated with nucleolar B23 [27]. To further investigate the association between sHBZ and B23, HEK293T cells were transfected with the myc-tagged sHBZ expression vector or the empty vector. Nucleoli were isolated from these transfected cells and different resulting fractions were subjected to immunoprecipitation with an anti-myc antibody. Western blot analysis with anti-B23 antibodies demonstrated the presence of B23 in these isolated fractions, as expected (Figure 3A). The results further indicated that sHBZ was associated with B23, predominantly within the nucleolus. To further evaluate this nucleolar association, similar co-immunoprecipitation experiments were conducted on cell fractions from C8166-45 T cells using an anti-HBZ antibody (Figure 3B). Western blot analysis revealed that B23 signals were again predominantly present in isolated nucleolar extracts. We finally assessed the sHBZ-B23 association in freshly infected cells. Following a previously established protocol [30], HeLa cells were infected with HTLV-1 by co-culturing with MT2 cells. As illustrated in Figure 3C, sHBZ was distinctly identified in infected HeLa cells, while it was absent in non-infected HeLa cells. Further examination revealed a pronounced association of B23 and sHBZ, particularly evident in the nucleolar extracts following the preparation of subcellular fractions and co-immunoprecipitation with anti-HBZ antibodies. The percentage of immunoprecipitated B23 levels from the detected input signal was next calculated and confirmed the more prevalent sHBZ-B23 association in the nucleolar fraction (Figure 3A–C). Overall, these findings compellingly demonstrate that sHBZ associates with B23 in HTLV-1-infected cells as well as in transfected cells, and, more significantly, within the nucleolar compartment.

## 4. Discussion

The HTLV-1 HBZ protein exists in two isoforms: the spliced isoform (sHBZ), which is the most abundant and arises from a spliced transcript, and the unspliced isoform (usHBZ) [20,21]. Previous studies have shown that sHBZ is partially localized in the nucleolus and plays a critical role in conferring immortalizing properties to T cells, whereas the non-nucleolar usHBZ isoform does not exhibit these properties [20,23]. Interestingly, the Antisense Protein of HTLV-2 (APH-2), the HBZ counterpart in the non-leukemogenic HTLV-2 virus, does not exhibit nucleolar localization and lacks immortalizing properties [32]. Based on these previous studies, we hypothesized that nucleolar localization of HBZ is a key feature underlying its immortalizing and transforming capacity, and that nucleolar-binding proteins are necessary regulators and mediators of this sHBZ-related function. In our recent study, we showed that sHBZ is associated with B23 [27], a nucleolar factor with multiple functions [28]. Herein, the objective of this study was to more precisely establish the existence of this association in the nucleolar compartment.

We first focused on isolating highly purified and enriched nucleoli from various cell types, including transfected HEK293T cells, chronically HTLV-1-infected T cells, and freshly infected HeLa cells. During the fractionation procedure, the integrity and purity of the nuclei, as well as the enrichment of the nucleolar fraction, confirmed that the nuclear fraction was devoid of other organelles. Western blot analysis confirmed the efficacy of the fractionation procedure, demonstrating that nucleolar-specific fibrillarin was exclusively present in the nucleolar fraction and absent from other cellular fractions.

Using our optimized nucleolar isolation method, we successfully demonstrated the nucleolar localization of sHBZ via Western blot analysis across all examined cell types. This result highlights that, in these three cell models, sHBZ is localized in the nucleolar compartment. In contrast, the unspliced isoform of HBZ (usHBZ) and APH2 were more abundantly detected in the cytoplasm and showed limited nucleolar localization, as previously reported [20,32]. Notably, our results differ from those of a recent study by Forlani et al., which reported that HBZ exhibits dual localization in both the nucleus and cytoplasm in ATL cells, with a stronger preference for cytoplasmic localization [33]. Another study from this research team suggested that HBZ was exclusively cytoplasmic in peripheral blood mononuclear cells (PBMCs) from asymptomatic carriers and from non-neoplastic pathologies [34]. A possible explanation for the observed discrepancy is that technical variability in the methodologies employed could influence the results. Differences in the sensitivity and specificity of the antibodies might account for variations in the detected localization patterns. The divergence between these findings underscores the complexity of HBZ biology and highlights the need for further studies to elucidate the factors dictating its subcellular distribution.

Purified nucleolar fractions, along with other cellular fractions, were utilized to investigate the sHBZ-B23 association through a co-immunoprecipitation approach. Our findings revealed that the sHBZ-B23 association predominantly occurs in the nucleolus. Although this complex was also detected in non-nucleolar compartments, our data strongly indicate that the nucleolus serves as the primary site of this association, highlighting its specificity within this compartmentalized cellular context.

The nucleolus is a critical sub-nuclear structure traditionally involved in ribosome biogenesis, but it also serves as a hub for several other cellular processes, including cell cycle regulation, stress response, and modulation of apoptosis [24,25]. This sub-nuclear compartment is a frequent target for viral proteins, which exploit its functions to facilitate viral replication and persistence [35]. Among the key nucleolar proteins, C23 (nucleolin) and B23 play pivotal roles in mediating the interplay between viral and host cellular machinery through their interactions with specific viral proteins [35,36,37]. The nucleolar localization of sHBZ and its association with B23 could suggest a mechanism employed by HTLV-1 to exploit critical nucleolar functions. In fact, several studies have specifically linked B23 to the functional regulation of the tumor suppressor proteins p53 and pRb [38,39,40]. Importantly, pRb has been implicated in the mechanism underlying HBZ-induced immortalization of T cells by modulating the activity of the E2F transcription factor, which in turn induces the expression of downstream target genes and promotes cell cycle progression [19]. It is plausible that the sHBZ-B23 complex may contribute to this process by sequestering pRb in the nucleolus, thereby enhancing E2F activation and facilitating cell cycle progression. We are, however, aware that these experiments were conducted in chronically/freshly infected cell lines, as well as transfected cells. Further experiments are required to confirm these data in ATL cell lines and PBMCs derived from infected individuals and to determine how this association could translate into the previously described properties attributed to this viral protein in the nucleolar context.

These insights emphasize the importance of understanding the molecular mechanisms underlying the sHBZ-B23 association. Determining how HTLV-1 manipulates nucleolar functions through the sHBZ-B23 complex should provide deeper insights into HTLV-1 pathogenesis and inform strategies for combating this persistent retrovirus.

## Data Availability

The raw data supporting the conclusions of this article will be made available by the authors on request.

## Supplementary Materials

The following supporting information can be downloaded at: https://www.mdpi.com/article/10.3390/v17050727/s1, Figure S1: Colocalization of C23 (nucleolin) and sHBZ in HeLa cells.

## References

[1] Poiesz B.J., Ruscetti F.W., Gazdart A.F., Bunnt P.A., Minnat J.D., Gallo R.C. (1980). Detection and Isolation of Type C Retrovirus Particles from Fresh and Cultured Lymphocytes of a Patient with Cutaneous T-Cell Lymphoma. Proc. Natl. Acad. Sci. USA.

[2] Gessain A., Cassar O. (2012). Epidemiological Aspects and World Distribution of HTLV-1 Infection. Front. Microbiol..

[3] Mortreux F., Gabet A.S., Wattel E. (2003). Molecular and Cellular Aspects of HTLV-1 Associated Leukemogenesis in Vivo. Leukemia.

[4] Yasunaga J.I. (2020). Strategies of Human T-Cell Leukemia Virus Type 1 for Persistent Infection: Implications for Leukemogenesis of Adult T-Cell Leukemia-Lymphoma. Front. Microbiol..

[5] Yamagochi K., Kiyokawa T., Nakada K., Yol L.S., Asou N., Ishii T., Sanada I., Seiki M., Yoshida M., Matutes E. (1988). Polyclonal Integration of HTLV-I Proviral DNA in Lymphocytes from HTLV-I Seropositive Individuals: An Intermediate State between the Healthy Carrier State and Smouldering ATL. Br. J. Haematol..

[6] Fujikawa D., Nakagawa S., Hori M., Kurokawa N., Soejima A., Nakano K., Yamochi T., Nakashima M., Kobayashi S., Tanaka Y. (2016). Polycomb-Dependent Epigenetic Landscape in Adult T-Cell Leukemia. Blood J. Am. Soc. Hematol..

[7] Matsuoka M., Jeang K.T. (2007). Human T-Cell Leukaemia Virus Type 1 (HTLV-1) Infectivity and Cellular Transformation. Nat. Rev. Cancer.

[8] Grassmann R., Aboud M., Jeang K.T. (2005). Molecular Mechanisms of Cellular Transformation by HTLV-1 Tax. Oncogene.

[9] Larocca D., Chao L.A., Seto M.H., Brunck T.K. (1989). Human T-cell leukemia virus minus strand transcription in infected T-cells. Biochem. Biophys. Res. Commun..

[10] Gaudray G., Gachon F., Basbous J., Biard-Piechaczyk M., Devaux C., Mesnard J.-M. (2002). The Complementary Strand of the Human T-Cell Leukemia Virus Type 1 RNA Genome Encodes a BZIP Transcription Factor That Down-Regulates Viral Transcription. J. Virol..

[11] Arnold J., Yamamoto B., Li M., Phipps A.J., Younis I., Lairmore M.D., Green P.L. (2006). Enhancement of Infectivity and Persistence in Vivo by HBZ, a Natural Antisense Coded Protein of HTLV-1. Blood.

[12] Satou Y., Yasunaga J.-I., Yoshida M., Matsuoka M. (2006). HTLV-I Basic Leucine Zipper Factor Gene mRNA Supports Proliferation of Adult T Cell Leukemia. Proc. Natl. Acad. Sci. USA.

[13] Hagiya K., Yasunaga J.I., Satou Y., Ohshima K., Matsuoka M. (2011). ATF3, an HTLV-1 BZip Factor Binding Protein, Promotes Proliferation of Adult T-Cell Leukemia Cells. Retrovirology.

[14] Basbous J., Arpin C., Gaudray G., Piechaczyk M., Devaux C., Mesnard J.M. (2003). The HBZ Factor of Human T-Cell Leukemia Virus Type I Dimerizes with Transcription Factors JunB and c-Jun and Modulates Their Transcriptional Activity. J. Biol. Chem..

[15] Thébault S., Basbous J., Hivin P., Devaux C., Mesnard J.M. (2004). HBZ Interacts with JunD and Stimulates Its Transcriptional Activity. FEBS Lett..

[16] Matsuoka M., Yasunaga J.I. (2013). Human T-Cell Leukemia Virus Type 1: Replication, Proliferation and Propagation by Tax and HTLV-1 BZIP Factor. Curr. Opin. Virol..

[17] Zhao T., Yasunaga J.-I., Satou Y., Nakao M., Takahashi M., Fujii M., Matsuoka M. (2009). Human T-Cell Leukemia Virus Type 1 BZIP Factor Selectively Suppresses the Classical Pathway of NF-κB. Blood.

[18] Wright D.G., Marchal C., Hoang K., Ankney J.A., Nguyen S.T., Rushing A.W., Polakowski N., Miotto B., Lemasson I. (2015). Human T-Cell Leukemia Virus Type-1-Encoded Protein HBZ Represses P53 Function by Inhibiting the Acetyltransferase Activity of P300/CBP and HBO1. Oncotarget.

[19] Kawatsuki A., Yasunaga J.I., Mitobe Y., Green P.L., Matsuoka M. (2016). HTLV-1 BZIP Factor Protein Targets the Rb/E2F-1 Pathway to Promote Proliferation and Apoptosis of Primary CD4+ T Cells. Oncogene.

[20] Murata K., Hayashibara T., Sugahara K., Uemura A., Yamaguchi T., Harasawa H., Hasegawa H., Tsuruda K., Okazaki T., Koji T. (2006). A Novel Alternative Splicing Isoform of Human T-Cell Leukemia Virus Type 1 BZIP Factor (HBZ-SI) Targets Distinct Subnuclear Localization. J. Virol..

[21] Cavanagh M.H., Landry S., Audet B., Arpin-André C., Hivin P., Paré M.È., Thête J., Wattel É., Marriott S.J., Mesnard J.M. (2006). HTLV-I Antisense Transcripts Initiating in the 3’LTR Are Alternatively Spliced and Polyadenylated. Retrovirology.

[22] Yoshida M., Satou Y., Yasunaga J., Fujisawa J., Matsuoka M. (2008). Transcriptional Control of Spliced and Unspliced Human T-Cell Leukemia Virus Type 1 BZIP Factor (HBZ) Gene. J. Virol..

[23] Matsuoka M., Green P.L. (2009). The HBZ Gene, a Key Player in HTLV-1 Pathogenesis. Retrovirology.

[24] Olson M.O., Dundr M., Szebeni A. (2000). The Nucleolus: An Old Factory with Unexpected Capabilities. Trend. Cell Biol..

[25] Pederson T. (1998). The Plurifunctional Nucleolus. Nucleic Acids Res..

[26] Maggi L.B., Weber J.D. (2005). Nucleolar Adaptation in Human Cancer. Cancer Investig..

[27] Liu Z., Larocque É., Xie Y., Xiao Y., Lemay G., Peloponese J.M., Mesnard J.M., Rassart É., Lin R., Zhou S. (2022). A Newly Identified Interaction between Nucleolar NPM1/B23 and the HTLV-I Basic Leucine Zipper Factor in HTLV-1 Infected Cells. Front. Microbiol..

[28] Box J.K., Paquet N., Adams M.N., Boucher D., Bolderson E., O’Byrne K.J., Richard D.J. (2016). Nucleophosmin: From Structure and Function to Disease Development. BMC Mol. Biol..

[29] Hivin P., Frédéric M., Arpin-André C., Basbous J., Gay B., Thébault S., Mesnard J.M. (2005). Nuclear Localization of HTLV-I BZIP Factor (HBZ) Is Mediated by Three Distinct Motifs. J. Cell Sci..

[30] Liu M., Yang L., Zhang L., Liu B., Merling R., Xia Z., Giam C.-Z. (2008). Human T-Cell Leukemia Virus Type 1 Infection Leads to Arrest in the G 1 Phase of the Cell Cycle. J. Virol..

[31] Chamousset D., Mamane S., Boisvert F.M., Trinkle-Mulcahy L. (2010). Efficient Extraction of Nucleolar Proteins for Interactome Analyses. Proteomics.

[32] Douceron E., Kaidarova Z., Miyazato P., Matsuoka M., Murphy E.L., Mahieux R. (2012). HTLV-2 APH-2 Expression Is Correlated with Proviral Load but APH-2 Does Not Promote Lymphocytosis. J. Infect. Dis..

[33] Forlani G., Shallak M., Tedeschi A., Cavallari I., Marçais A., Hermine O., Accolla R.S. (2021). Dual Cytoplasmic and Nuclear Localization of HTLV-1-Encoded HBZ Protein Is a Unique Feature of Adult T-Cell Leukemia. Haematologica.

[34] Baratella M., Forlani G., Raval G.U., Tedeschi A., Gout O., Gessain A., Tosi G., Accolla R.S. (2017). Cytoplasmic Localization of HTLV-1 HBZ Protein: A Biomarker of HTLV-1-Associated Myelopathy/Tropical Spastic Paraparesis (HAM/TSP). PLoS Negl. Trop. Dis..

[35] Greco A. (2009). Involvement of the Nucleolus in Replication of Human Viruses. Rev. Med. Virol..

[36] Lobaina Y., Perera Y. (2018). Implication of B23/Nucleophosmin in Viral Infections, Potential Uses of B23/NPM1 Inhibitors as Antiviral Therapy. Infect. Disord. Drug Targets.

[37] Zakaryan H., Stamminger T. (2011). Nuclear Remodelling during Viral Infections. Cell Microbiol..

[38] Takemura M., Ohoka F., Perpelescu M., Ogawa M., Matsushita H., Takaba T., Akiyama T., Umekawa H., Furuichi Y., Cook P.R. (2002). Phosphorylation-Dependent Migration of Retinoblastoma Protein into the Nucleolus Triggered by Binding to Nucleophosmin/B23. Exp. Cell Res..

[39] Lindström M.S., Zhang Y. (2006). B23 and ARF: Friends or Foes?. Cell Biochem. Biophys..

[40] Liu X., Liu Z., Jang S.-W., Ma Z., Shinmura K., Kang S., Dong S., Chen J., Fukasawa K., Ye K. (2007). Sumoylation of Nucleophosmin/B23 Regulates Its Subcellular Localization, Mediating Cell Proliferation and Survival. Proc. Natl. Acad. Sci. USA.

