# Supplementary material for: HTLV-I Basic Leucine Zipper Factor (sHBZ) Actively Associates with Nucleophosmin (B23) in the Nucleolus
Source: Viruses. 2025 May 19;17(5):727. doi: 10.3390/v17050727 (PMC12115704; doi:10.3390/v17050727)
Supplement: Supplementary file 1 [file viruses-17-00727-s001.zip › viruses-3550205-supplementary.pdf]

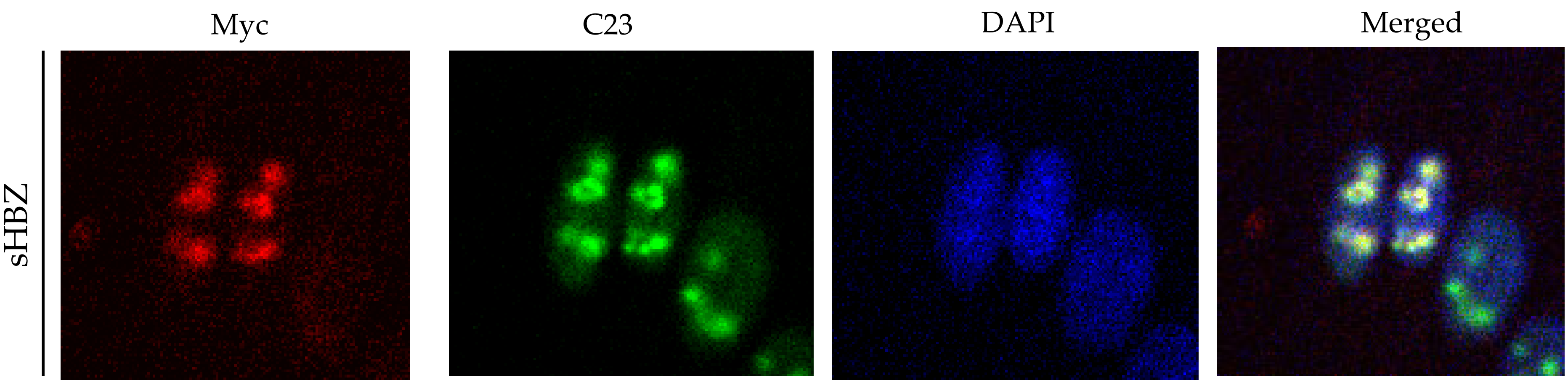

Supplementary Figure 1: Colocalization of C23 (nucleolin) and sHBZ in HeLa cells. HeLa cells were transfected with expression vectors for myc-tagged sHBZ and GFP-fused sHBZ. Fixed cells were analyzed by confocal microscopy with anti-C23 and anti-myc antibodies following staining with DAPI.
